# The Role of Fundamental Movement Skills and Spatial Abilities in the Relationship between Physical Activity and Mathematics Achievement in Primary School Children

**DOI:** 10.3390/jintelligence12020022

**Published:** 2024-02-16

**Authors:** Jessica Scott, Tim Jay, Christopher M. Spray

**Affiliations:** 1 School of Sport, Exercise and Health Sciences, Loughborough University, Loughborough LE11 3TU, UK; 2 School of Education, University of Nottingham, Nottingham NG7 2RD, UK

**Keywords:** physical activity, mathematics achievement, fundamental movement skills (FMS), spatial ability

## Abstract

Research has demonstrated positive relationships between fundamental movement skills (FMS) and mathematics achievement in children, and this relationship may be mediated by spatial ability. Engaging in physical activity (PA) may also have positive outcomes on mathematics achievement; however, no study has investigated this network of relationships together. This study aimed to examine the relationship between PA and mathematics achievement, and the mediating effects of FMS and spatial abilities, in primary school children. Using a cross-sectional design, data were collected from 182 children (aged 7 to 8 years old) across four schools in England. Objective moderate-to-vigorous PA (MVPA) levels and subjective parental reports of their children’s PA participation were collected. Children’s FMS were assessed, along with their performance on four spatial ability tasks and a mathematics test. Mediation analyses revealed no significant mediation effects of FMS and spatial abilities on the positive significant relationship between MVPA and mathematics achievement; however, spatial ability partially mediated the relationship between FMS and mathematics achievement. These results suggest that FMS and spatial ability may not be related to MVPA in this network of relationships, but children with more mature FMS perform better in mathematics due to them performing better on specific spatial ability tasks.

## 1. Introduction

Physical activity (PA) is defined as bodily movement produced by skeletal muscles that requires energy expenditure (Caspersen et al. 1985; WHO 2018). It is well established that PA makes an important contribution to the holistic development of children. PA improves children’s physiological (Janssen and LeBlanc 2010), physical (Poitras et al. 2016), and psychological (Biddle and Asare 2011) wellbeing. However, there is much debate about the effects PA may have on school-centered academic outcomes. There is a growing body of research investigating the effects of PA (curriculum-integrated, school-based, extracurricular, and out-of-school PA) on academic achievement; however, the findings are inconclusive. This research study aimed to examine the specific relationship between PA and mathematics achievement, and the mediating effects that fundamental movement skills (FMS) and spatial abilities may have on this relationship, in primary school children.

Research suggests there is a positive association between PA and academic achievement, specifically performance in mathematics. Rasberry et al. (2011) found that half of the 50 studies analyzed in their systematic review showed a positive correlation between school-based PA and mathematics achievement in children and adolescents. In more recent systematic reviews, Sneck et al. (2019) and James et al. (2023) examined PA interventions in relation to academic achievement in children and adolescents and found positive effects, and no harmful or negative effects, of PA interventions on mathematics achievement. Not all studies examined found positive effects of PA on mathematics achievement; several studies found insignificant effects of PA on mathematics. For example, there was no significant effect found on mathematics performance in 10-to-11-year-olds who completed a 7-month PA intervention, which increased PA by participating in 90 min of physically active education lessons in the school playground, having 5 min PA breaks during classroom lessons, and completing 10 min a day of PA homework each week compared to children in the control group who received the normal school curriculum (Resaland et al. 2016). Despite mixed findings in the literature, there is still strong enough evidence to conclude that there are beneficial effects from taking part in PA on mathematical achievement in children (Singh et al. 2019), with the evidence highlighting that PA is not detrimental to mathematical achievement in children and may even enhance it.

Fundamental movement skills (FMS), comprising basic locomotion, object manipulation, and stabilization skills (Rudd et al. 2015), may be related to both PA and mathematics achievement. Stodden et al. (2008) devised a conceptual model that suggests that the relationship between PA and FMS is bidirectional. Based on limited research, the model suggested that PA is associated with motor competence development in early childhood, whereas motor competence is predictive of PA in mid and late-childhood. Since this conceptualization, research has found positive relationships between PA and motor development in mid-childhood; FMS are improved with greater PA exposure in primary school students. Ericsson (2011) found that FMS improved after being exposed to more PA from the age of 7 to 15 years old, with the beneficial effects of PA on FMS being observed after just one year, compared to children in the control group. In addition, Dapp et al. (2021) found that engaging in structured PA, either exclusively or in combination with unstructured PA, resulted in better FMS development in children aged 6 to 8 years old, and Melby et al. (2021) found that diverse PA at 6 years old was significantly associated with greater motor competence at age 9 to 13 years, but there was no longitudinal association found from motor competence at 6 years old to PA engagement at 9 to 13 years old. Therefore, it appears that there is a positive and potential predictive association between PA and FMS in children.

There is evidence to suggest a positive relationship between FMS and mathematics achievement in children. Macdonald et al.’s (2018) systematic review concluded that there was strong evidence to support a positive association between total FMS proficiency and mathematical achievement; however, this is a very-weak-to-moderate association. With regards to preschool-aged children, improvements in 3 to 5-year-olds’ motor competence skills were positively related to improvements in their mathematics problem-solving skills (Willoughby et al. 2021). In children aged 5 to 6 years old, de Waal (2019) found that locomotion and stability FMS were positively correlated with numerical and geometrical mathematical abilities, but no significant correlations were found between object manipulation skills and mathematics achievement. In slightly older primary school-aged children, Scott et al. (2023) found that total locomotion score (as assessed by running, hopping, and jumping) and overall FMS score were weakly positively related to total mathematics scores, supporting the conclusions made in Macdonald et al.’s (2018) systematic review. This highlights that FMS proficiency may be beneficial for mathematical achievement and therefore may mediate the relationship between PA and mathematics.

Spatial ability may also play a role in this network of relationships. Research is beginning to reveal that PA and FMS may positively relate to spatial ability in children. Moraweitz and Muehlbauer (2021) state that the literature shows a potential for spatial abilities to be improved in children and adolescents by participating in PA. When investigating the relationship between PA and FMS with specific spatial abilities, much research focuses on mental rotation performance, which is an intrinsic–dynamic spatial ability (Jansen and Richter 2015; Pietsch et al. 2017), ignoring the other three spatial abilities described by Uttal et al. (2013). These authors classified spatial skills as being either intrinsic or extrinsic (the ability to describe the spatial characteristics of objects either individually or in relation to one another, respectively) and dynamic or static (the ability to define these characteristics requires either physical or mental transformation or not, respectively), resulting in four spatial abilities: intrinsic–static (IS), intrinsic–dynamic (ID), extrinsic–static (ES), and extrinsic–dynamic (ED). Two recent studies examined the relationships between FMS and all four of the spatial abilities described in primary school children. Running and throwing were positively related to IS and ES spatial abilities, respectively, whereas no significant relationship was found between jumping and ID spatial ability (Jansen and Pietsch 2022), and locomotion, object manipulation, and total FMS scores were positively related to IS spatial ability (Scott et al. 2023).

The evidence highlights an established relationship between spatial ability and mathematics skills. This relationship has been examined since the early 20th century and is consistent in older children and adults, but there is a lack of research examining the relationship in children below the age of 10 years (Mix and Cheng 2012). However, recently, Atit et al. (2022) found that there was a positive moderate association between spatial skills and mathematics achievement in preschool, primary, and secondary school children in their systematic review. Specifically, IS, ID, ES, and ED spatial abilities positively correlated with mathematics achievement, as assessed by numeracy, arithmetic, geometry, and logical reasoning skills in children (Gilligan et al. 2019; Xie et al. 2020). Scott et al. (2023) examined the mediating effects of spatial ability on the relationship between FMS proficiency and mathematics achievement and found that IS spatial ability fully mediated this relationship. ED spatial ability did not positively relate to FMS and the remaining spatial abilities were not included in the analysis; however, this research begins to highlight the important part that spatial ability plays in this network of relationships, and further research is needed to confirm the effect that spatial ability may have on the relationship between PA and mathematics. Therefore, spatial ability and FMS should also be examined when exploring the relationships between PA and mathematics achievement in children.

The present study aims to contribute to the growing body of literature examining the factors that may positively influence achievement in mathematics in young children, specifically, the effects that PA, FMS, and spatial abilities have on mathematical achievement. Previous research has individually examined the impact that these factors have on performance in mathematics; however, there is a distinct gap in the literature when it comes to understanding the interrelationships among these factors. We address this gap by conducting a comprehensive examination into how PA, FMS, and spatial abilities are related and collectively contribute to mathematical achievement in young children. This research examines both objective and subjective PA in relation to mathematics achievement, which is assessed through performance in numeracy, arithmetic, and geometry. This research also examines the mediating effects that total FMS proficiency (as assessed by locomotion, object manipulation, and stabilization skills) and spatial ability (as assessed by age-appropriate tests of IS, ID, ES, and ED spatial ability) have, building upon the methodological limitations of previous research. Based on the evidence reviewed, we hypothesized that (H1) PA will positively associate with mathematics achievement; and (H2) FMS proficiency and spatial ability will mediate the relationship between PA and mathematics achievement.

## 2. Materials and Methods

### 2.1. Participants and Procedure

Two-hundred-and-eight Year 3 children were recruited from four UK primary schools. Information packs were sent to parents/guardians via school channels to provide opt-in parental consent. Children also provided their informed assent. Using a cross-sectional correlational design, data collection on 182 children (85 boys, mean age: 8.19, *SD* = .314) took place from April to July 2023. This meets the required sample size needed for a mediation analysis when conducting a mediation analysis using the bootstrapping (bias-corrected) method. For an anticipated effect size of .26, for both paths a and b, a statistical power of 80%, and a probability level of .05, a minimum of 148 participants is needed (Fritz and MacKinnon 2007). At each school, FMS were assessed during a PE lesson, taking approximately one hour to assess 30 children. On a separate day, each child completed a mathematics test during a mathematics lesson, taking approximately 45 min to complete. The following week, at the start of the school day, the children were given an accelerometer to wear on their wrist for 7 consecutive days (from Monday to Sunday). During this week, each child also independently completed one computer and three paper-and-pen tasks assessing spatial ability, taking approximately 30 min to complete. Each child also completed a verbal intelligence task, taking 5 min to complete. Throughout this period, parents responded to an online questionnaire to report the sport and leisure activities their child usually participated in, which took approximately 10 min to complete.

### 2.2. Measures

#### 2.2.1. FMS

FUNMOVES (Eddy et al. 2021), a class-level product measure, assessed the locomotion, object manipulation, and stabilization skills of the children (see Eddy et al. (2021) and Scott et al. (2023) for full procedural description). For this research, a total score for FMS was computed by combining the total locomotion score (running, jumping, and hopping scores), total object manipulation score (throwing and kicking scores), and stability score (balance score) together. There is no official total score that can be obtained due to the running assessment; however, children can score a maximum of 4 for the jumping, hopping, and balance skills, a maximum score of 10 for the throwing skill, and a maximum score of 5 for the kicking skill.

#### 2.2.2. Mathematics Achievement

To assess mathematics achievement, the Mathematics Assessment for Learning and Teaching 8 (MaLT8; Williams 2005) was used (α = .90), which is in line with the English national curriculum for Year 3, where children are aged 7 to 8 years old (Williams 2005). For the present study, numerical ability (total score of 21, α = .83) was measured through the counting, understanding numbers, and knowing and understanding number facts questions. Arithmetical ability (total score of 10, α = .71) was measured through the calculation questions, and geometrical ability (total score of 14, α = .69) was measured through the understanding shape, measurement, and handling data questions. A total score of 45 could be obtained.

#### 2.2.3. PA

Objective Moderate-to-Vigorous PA (MVPA) Levels. Objective MVPA was assessed using GT9X accelerometers (ActiGraph, Pensacola, FL, USA). Participants were required to wear the monitor on their wrist for seven consecutive days, including the weekend, except for sleeping and water-based activities. There was no display activated on the accelerometer screen. Due to the sporadic nature of children’s movements, more frequent assessment is required (Bailey et al. 1995). The current study collected the data at a sampling frequency of 100 Hz. The raw data were downloaded after completion using ActiLife (version six, Pensacola, FL, USA). Data were deemed valid if the children wore the monitors for a minimum of 10 h for 2 days (Rich et al. 2013). The average total number of minutes spent undertaking MVPA per day was then calculated; it does not provide information about what specific activities children participated in.

Parent-reported PA. Parents/guardians completed a modified version of the proxy Children’s Leisure Activities Study Survey (CLASS; Telford et al. 2004). The questionnaire included a checklist of 23 physical activities (e.g., dance, football, walking to and from school, and household chores) and 14 leisure activities (e.g., watching TV, reading, and completing homework). Parents/guardians were required to indicate whether their child participates in each activity, and if they answer yes, they must report the frequency of the activity and the time spent participating in the activity separately for Monday to Friday and on the weekend. The total number of hours spent undertaking physical activities during a typical week was calculated.

#### 2.2.4. Spatial Ability

IS Spatial Ability. The paper-and-pencil Children’s Embedded Figures Task (Witkin et al. 1971) assessed IS spatial ability (α = .80). Children had to disembed a complex picture to locate a tent shape for 11 trials, and a house shape for 14 trials. One point was awarded for each trial where the shape was correctly identified, resulting in a possible total score of 25.

ID Spatial Ability. The paper-and-pencil animal stimuli Picture Mental Rotation Task (Neuburger et al. 2011) assessed ID spatial ability (α = .86). Children had two minutes to complete 16 trials where they had to select the two rotated pictures from the four presented that matched the target item. The other two were rotated mirror images. One point was awarded if both correct pictures were selected, resulting in a possible total score of 16.

ES Spatial Ability. Gilligan et al.’s (2018) computerized spatial scaling task assessed ES spatial ability (α = .62). Throughout 18 trials, children were shown model pirate maps, either a 6 × 6 or 10 × 10 square, on an A3 flipchart. Children had to then select the corresponding referent map presented from a selection of four shown on a computer screen, which varied in scaling factor every six trials (same size, half the size, and a quarter of the size of the model map). One point was awarded if the correct referent map was selected, resulting in a possible total score of 18.

ED Spatial Ability. The paper-and-pencil Perspective Taking Task (Frick 2019) assessed ED spatial ability (α = .82). Children were presented with 22 pictorial representations of objects and Lego characters and had to select which of the four pictures was the correct photo taken by the character holding the camera in the pictorial representation. The trials differed in complexity and angular difference between the child and the photographer. One point was awarded if the correct photo was identified, resulting in a possible total score of 22.

#### 2.2.5. Control Variables

The Word Reading A Subscale of the British Ability Scales III (BAS-III, Elliot and Smith 2011) was administered to control for intelligence. Participants were asked to read aloud words shown to them. One point was awarded for each correctly phonetically read word, resulting in a possible total score of 90. This measure has previously been used in research that examines the relationship between spatial ability and mathematics to control for reading ability in children (Gilligan et al. 2019). As reading ability is highly correlated with general intelligence (Carver 1990), this subscale was used as a measure of intelligence.

### 2.3. Data Analysis

Calculations for means, standard deviations, and Spearman correlations were performed using IBM SPSS 28.0. Partial Spearman correlations were also completed, controlling for verbal intelligence. A mediation analysis, whilst controlling for verbal intelligence, was completed using the “Specific Indirect Effects” estimand in SPSS AMOS 26 (Gaskin et al. 2020) using 5000 bias-corrected bootstrapped samples, and significant indirect effects were considered if 95% confidence intervals did not include zero (Williams and MacKinnon 2008). Hildebrand et al.’s (2014, 2016) PA cut-off points from the accelerometer raw data were analyzed using the GGIR R package for accelerometry (Migueles et al. 2019). The cut-off points are 35.6, 201.4, and 707.0 for light, moderate, and vigorous PA, respectively.

## 3. Results

### 3.1. Preliminary Analysis

All 182 participants completed the FMS, spatial ability, mathematics, and verbal intelligence assessments (85 males, *M* age = 8.19, *SD* age = .314). Of the 182 children, 139 returned parental responses from the CLASS to assess subjective PA. Five responses were identified as extreme outliers and removed, resulting in a total of 134 participants with parental-reported PA data (61 males, *M* age = 8.18, *SD* age = .311). With regards to objective MVPA, the criterion of 10 h minimum wear time for 2 days was appropriately selected for this research (Rich et al. 2013), and resulted in 137 children with validated accelerometer data (63 males, *M* age = 8.20, *SD* age = .322). The majority of the data for FMS, spatial ability, mathematics, and verbal intelligence were not normally distributed (D_(182)_ ≥ .082, *p* ≤ .004, with the exception of IS spatial ability, for which scores were normally distributed: D_(182)_ = .066, *p* = .052). The data for objective MVPA and subjective PA were also not normally distributed (D_(137)_ = .081, *p* = .028, and D_(134)_ = .126, *p* < .001, respectively).

### 3.2. Descriptive Statistics and Correlations

On average, participants scored 65% and over on all of the composite scores for FMS, performing best in locomotion skills. Participants scored, on average, over 60% on all of the spatial ability tests, except for IS spatial ability (mean score 13.45 out of 25), with participants performing the best on the PTT. Participants, on average, scored above 50% on all of the mathematical abilities, with performance in numerical ability being the best and performance in geometrical ability being the worst, and the participants, on average, scored 80% in the verbal intelligence assessment. With regards to objective MVPA, on average, the children completed just over 80 min a day of MVPA.

Spearman correlation coefficients were calculated across the whole sample (*n* = 182) to examine the interrelationships between PA, FMS proficiency, spatial ability, and performance in mathematics to test H1 (see Table 1). Neither objective MVPA nor subjective PA were significantly correlated with total mathematics score (rs = .108, *p* = .207, and rs = .057, *p* = .511, respectively), nor any of the individual mathematical abilities. Verbal intelligence significantly positively correlated with all spatial and mathematical abilities; therefore, partial Spearman correlation coefficients were calculated to examine these relationships whilst controlling for verbal intelligence across the whole sample (*n* = 182) (see Table 2). When verbal intelligence was controlled for, objective MVPA was significantly positively correlated with numerical ability (rs = .211, *p* = .014), arithmetical ability (rs = .199, *p* = .020), and total mathematics score (rs = .178, *p* = .038), but did not correlate with total FMS score or any of the four spatial abilities. The relationship between subjective PA and mathematics achievement remained non-significant (rs = .094, *p* = .284). Further to this, when controlling for verbal intelligence, total FMS score significantly correlated positively with spatial ability and mathematics achievement, and all four spatial abilities significantly correlated positively with mathematics achievement. Specifically, total FMS score positively correlated with IS and ES spatial abilities (rs = .312, *p* < .001, and rs = .227, *p* = .002, respectively), total mathematics achievement (rs = .295, *p* < .001), and numerical, arithmetical, and geometrical abilities.

### 3.3. Mediation Analysis

**Figure 1:**
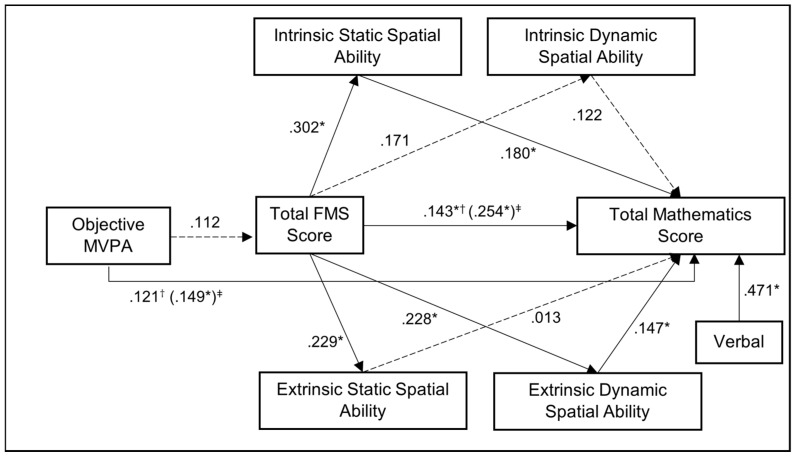
Objective MVPA mediation model controlling for verbal intelligence (*n* = 137). Note: standardized coefficients reported. * *p* < .05. † Standardized direct effect coefficient. ‡ Standardized total effect coefficient.

As a significant association was found between objective MVPA and total mathematics score whilst controlling for verbal intelligence (β = .030, 95% CI [.002 to .055]), a mediation analysis of the effect of FMS and spatial ability on this relationship, whilst controlling for verbal intelligence, was conducted on the sample with validated MVPA data (*n* = 137), testing H2. Total FMS score and all four spatial abilities were inputted into the model (χ^2^_(11)_ = 82.114, *p* < .001). The direct effect of MVPA on mathematics achievement in the presence of all of these variables was not significant (β = .024, 95% CI [−.001 to .048]), suggesting that some of the indirect effects of MVPA on mathematical achievement through the FMS and spatial ability mediation pathways might be significant. With regards to FMS, path a (i.e., MVPA → FMS) was not significant (β = .008, 95% CI [−.004 to .021]), but path b (i.e., FMS → mathematics achievement) was significant (β = .379, 95% CI [.078 to .697]); however, the indirect effect of MVPA on mathematics achievement through FMS was not significant (β = .003, 95% CI [−.001 to .012]), highlighting no mediation. The serial mediation pathways of FMS and each of the four spatial abilities on the relationship between MVPA and mathematics achievement revealed significant indirect effects between time spent undertaking MVPA and mathematics achievement through FMS and IS spatial ability (β = .001, 95% CI [.000 to .005]), FMS and ID spatial ability (β = .000, 95% CI [.000 to .003]), and FMS and ED spatial ability (β = .000, 95% CI [.000 to .005]). No significant indirect effect was found for the relationship between MVPA and mathematics achievement through FMS and ES spatial ability (β = .000, 95% CI [−.001 to .002]); see Figure 1. However, we cannot confirm these mediation effects that derive from the serial pathway from FMS proficiency to spatial ability on the relationship between MVPA and mathematics score, as the direct effect between objective MVPA and total FMS score was not significant (β = .008, 95% CI [−.004 to .021]).

The model also highlights that IS spatial ability and ED spatial ability partially mediated the relationship between FMS score and mathematics achievement. The indirect effects of IS and ED spatial ability on the relationship between FMS proficiency and mathematics achievement were significant (β = .144, 95% CI [.031 to .311], and β = .089, 95% CI [.005 to .274], respectively), whereas the indirect effects of ID and ES spatial ability on this relationship were not significant (β = .055, 95% CI [−.004 to .201], and β = .008, 95% CI [−.086 to .104], respectively). As both the total and direct effects from total FMS score to mathematics achievement were significant (β = .675, 95% CI [.288 to 1.028], and β = .379, 95% CI [.078 to .697], respectively), these results highlight partial mediation. No mediation analyses were completed for the effects of FMS and spatial ability on the relationship between subjective PA and mathematics achievement as there was no significant relationship found between subjective PA and achievement in mathematics.

## 4. Discussion

The present study examined the relationship between PA and mathematics achievement in 7-to-8-year-old children, and the mediating role of FMS and spatial ability on this relationship. The results highlight that when controlling for verbal intelligence, time spent undertaking MVPA per day was significantly positively correlated with mathematics achievement; however, the number of hours children spent undertaking PA a week, as reported by their parents, was not significantly related with mathematics, providing partial support for H1. Support for H2 was not found. There was no significant indirect effect of FMS proficiency on the relationship between MVPA and mathematics achievement, and despite weak significant indirect effects being found for the pathways from FMS to IS spatial ability, FMS to ID spatial ability, and FMS to ED spatial ability on the relationship between time spent undertaking MVPA per day and mathematics achievement, mediation cannot be concluded due to the insignificant relationship between MVPA and FMS proficiency.

### 4.1. PA and Mathematics Achievement

When controlling for verbal intelligence, a weak but significant positive relationship was found between average time spent undertaking MVPA per day and total mathematics score, specifically with numerical and arithmetical abilities. This finding is supported by the current literature which has found improved academic achievement, specifically achievement in mathematics, in children and adolescents after engaging in PA (de Bruijn et al. 2020; James et al. 2023). However, initial correlations, when not controlling for verbal intelligence, did not show a significant association between MVPA and total mathematics score. This suggests that the benefits of MVPA on mathematical skills might be masked when not accounting for individual differences in verbal intelligence, highlighting the importance of considering verbal intelligence when examining this relationship.

On the other hand, subjective PA did not show a significant relationship with mathematics achievement, irrespective of controlling for verbal intelligence. Despite previous research suggesting that parental reports of children’s PA do provide reliable estimates of children’s PA (Telford et al. 2004), our results only highlight a weak relationship between parental reports of their children’s PA levels and objective MVPA. Our findings may represent the limitations of proxy measures to assess children’s PA levels. Children’s PA is not just accounted for by the different physical and sporting activities that they participate in, but also through spontaneous and sporadic free play and movement throughout the day (Welk et al. 2000). Also, parents may have few opportunities to observe all of the physical activities their child participates in, and social desirability is also likely to occur. This finding indicates that the proxy-reported data of children’s PA levels may not be as reliable and objective as MVPA levels, determined by accelerometry, to predict mathematics achievement in young children. Overall, the results of objectively measured PA contribute to the literature, providing support for the notion that objectively measured MVPA is positively associated with mathematics achievement, potentially providing a beneficial effect in children (Singh et al. 2019), highlighting that children who participate in more MVPA per day may achieve higher mathematics scores.

Possible explanations for the positive association between MVPA and mathematics achievement have been proposed. Research suggests that higher intensity PA may result in improved aerobic fitness (Hannan et al. 2018), which is a known predictor of academic achievement (Donnelly et al. 2016). Aerobic fitness creates short-term changes, such as increased cerebral blood flow and upregulation of BDNF, which result in the development of new blood vessels and neurons and an increase in synaptic plasticity, creating long-term changes in the brain regions responsible for learning, such as the prefrontal cortex (Best 2010), thus aiding in improving academic achievement. Furthermore, the theory of embodied cognition proposes that mathematics achievement may be aided by participation in PA due to sensorimotor experiences grounding mathematical concepts, making them more meaningful and understandable (Lakoff and Núñez 2000).

### 4.2. The Mediating Role of FMS Proficiency and Spatial Abilities

No significant relationship was found between MVPA and total FMS score. This refutes research in this area that highlights not only cross-sectional but also longitudinal and experimental research supporting a positive association between PA and motor skills in preschool children, primary school children, and adolescents (Holfelder and Schott 2014; Jones et al. 2020; Logan et al. 2015; Morgan et al. 2013). Previous research has also found a significant directional relationship between the two factors in mid-childhood (Melby et al. 2021), which was undetermined by our results. This difference may be due to our measurement of MVPA, which may capture PA in both unstructured and structured environments. Dapp et al. (2021) found that engaging exclusively in unstructured PA does not benefit motor skill development; participation in exclusively structured PA or in combination with unstructured PA was positively associated with FMS. Our accelerometer data did not distinguish between structured and unstructured PA, so perhaps some of the data were solely based on unstructured PA, which may explain the lack of relationship found between MVPA and FMS proficiency. Furthermore, the accelerometer data were not validated to the most stringent parameters. Valid data were calculated as having a minimum wear time of 10 h for any two days of the week, whereas other research involving children required a minimum wear time of 10 h for three weekdays and one weekend day (Nagy et al. 2019). Therefore, this more constrained data might also explain why no relationship was found between MVPA and FMS.

The results of this study also support previous research that has found a positive relationship between FMS and mathematics achievement in children. Specifically, a significant positive but weak relationship was found between total FMS score and all areas of mathematics and total mathematics score, supporting previous research also conducted with primary school-aged children (de Bruijn et al. 2019; de Waal 2019; Scott et al. 2023). These results are aligned with the conclusion that there is strong evidence for a weak-to-moderate relationship between FMS proficiency and mathematics achievement (Macdonald et al. 2018). The results of this study also found positive relationships between FMS and spatial ability, and spatial ability and mathematics achievement. A significant positive, but also weak, relationship was found between total FMS score and IS and ES spatial ability. This finding is supported by Scott et al. (2023), who identified that total FMS score was weakly positively correlated with IS spatial ability but was not associated with ED spatial ability. However, it is surprising that total FMS score did not significantly correlate with ID spatial ability, as previous research has found that FMS, specifically object manipulation skills, are positively associated with performance in mental rotation tasks, an ID spatial ability (Jansen et al. 2011). This inconsistency may be accounted for by the different mental rotation tasks being used. Jansen et al. (2011) used a more complex mental rotation task involving 3D block figures and only assessed this on girls’ juggling performance, a complex object manipulation skill, whereas the current research used 2D animal stimuli and assessed the performance on this skill in relation to an accumulation of one’s performance in all three FMS constructs. In addition, the results highlight that all four spatial abilities were significantly positively related, albeit weakly, with all three of the mathematics abilities assessed and total mathematics score, which is in line with the findings from recent systematic reviews and meta-analyses (Atit et al. 2022; Xie et al. 2020). The results of the present study add support to this field of research, suggesting that spatial ability may play a role in the relationship between FMS and mathematics achievement, and potentially the relationship between MVPA and mathematics achievement.

With regards to the mediation analysis, the finding that there was no relationship between MVPA and FMS explains why the mediation analysis revealed no effect of MVPA on mathematics achievement through FMS proficiency. This suggests that FMS may not mediate the relationship between PA and mathematics achievement. When incorporating spatial abilities in the mediation model, the mediation analysis found significant effects of spatial ability on the relationship between MVPA and mathematics achievement through FMS. However, as there was no positive relationship between time spent undertaking MVPA per day and total FMS score, we cannot conclude that spatial cognition is a mediator in the relationship between MVPA and mathematics achievement. This finding was somewhat unexpected, as existing research suggests a potential mediating role of motor skills in the relationship between PA and mathematics achievement (Melby et al. 2021). Lopes et al. (2013) argue that children should participate in PA that promotes motor skill development due to the important role that FMS have on academic achievement. In the future, PA that is conducted in structured settings or specifically promotes motor skills should be assessed, which our assessment of PA levels did not incorporate, to better understand the network of relationships between PA, FMS, and mathematics achievement.

However, the model highlights that spatial ability may be a potential mediator in the relationship between FMS proficiency and mathematics achievement. Children with more proficient overall FMS may have scored higher on their mathematics assessment in part due to their motor skills helping them develop their spatial abilities, which aid in mathematics learning. Specifically, IS and ED spatial ability partially mediated the positive relationship between total FMS score and total mathematics score, which supports previous research conducted with children of a similar age (Scott et al. 2023). Children with more proficient FMS may perform better on tests of IS spatial ability due to their motor expertise of detecting visual cues efficiently in their sporting or physical activity environment in order to perform successfully, enhancing their ability to extract information by ignoring distracting stimuli and focusing on the target (Voss et al. 2010). This skill is important for improving their mathematics learning as it allows individuals to discriminate between place values and “pull apart” figures and shapes (Nilges and Usnick 2000). In addition, children with more proficient FMS may perform better on tests of ED spatial ability due to their motor expertise of working with others in a team and against opponents to perform successfully, enhancing their ability to recognize other people’s perspectives, which aids with understanding calculations and patterns in mathematics learning (Nilges and Usnick 2000). In sum, spatial abilities may be important in explaining the relationship between FMS proficiency and mathematics achievement; however, FMS proficiency and performance in spatial ability are not mediators and explanatory factors for the relationship between time spent undertaking MVPA per day and mathematics achievement.

### 4.3. Limitations and Future Directions

This is the first study to examine the network of relationships between PA, FMS, spatial abilities, and mathematics in one study, but it is not without its limitations. The cross-sectional design limits the ability to establish causal relationships. Furthermore, mediational findings based on cross-sectional data should be treated with caution (Maxwell et al. 2011). It is justified to use this analytical method when theoretically informed models are tested and real-world practical constraints in schools mean that data collection schedules are not always under the researcher’s control (Cain et al. 2018). Nevertheless, the current findings should be replicated with longitudinal temporally sequenced data from larger samples so that the influence of residual change in variables can be tested within the hypothesized network of relationships and greater confidence regarding directional influence can be provided. Moreover, the manipulation of PA or FMS could examine causal effects on spatial abilities and mathematics in controlled experimental trials. Such knowledge would be beneficial for primary school teachers to help guide their PE sessions, ensuring they are delivering the recommended PA guidelines in school and make structured PA accessible for children from all socio-economic backgrounds, and to improve mathematics achievement across the board.

Further, the spatial scaling task to assess ES spatial ability has low internal consistency, and therefore, caution must be taken with the relationships found in this network with regards to ES spatial ability. The current study also had a relatively small sample size after validating the accelerometer data, which future research should address to ensure that the sample size meets the power analysis for the mediation analysis and to enhance the confidence of these findings. In addition, future research should increase the size and age range of the sample to examine developmental changes in this network of relationships. The present study highlights that MVPA positively correlates with numerical and arithmetical, but not geometrical, achievement. Future research could identify if these relationships remain the same, or if a positive relationship with more sophisticated mathematical skills, such as geometry, is found, and whether spatial relations would begin to play a role as children become older.

## 5. Conclusions

In conclusion, our study contributes to the understanding of the complex relationships between PA, FMS, spatial abilities, and mathematics achievement in primary school children. Objectively measured MVPA levels per day appear to be positively associated with mathematics achievement when accounting for individual differences in verbal intelligence. FMS and spatial abilities do not play a mediating role in this relationship due to the lack of relationship between MVPA and FMS proficiency; however, specific spatial abilities may explain part of the relationship found between overall FMS proficiency and mathematics achievement. These results, however, should be regarded with caution, pending further research that overcomes the limitations of the current study to provide further insight into this network of relationships.

## Figures and Tables

**Table 1:** Spearman correlations between PA, FMS, spatial abilities, verbal intelligence, and mathematics in the whole sample (*n* = 182).

Variable	Mean (SD)	1	2	3	4	5	6	7	8	9	10	11	12	13	14
1. Total Locomotion	13.23 (1.753)	-													
2. Total Object Manipulation	10.10 (1.686)	.209 **	-												
3. Stability	2.58 (.880)	.260 **	.097	-											
4. Total FMS Score	25.91 (3.034)	.789 **	.673 **	.487 **	-										
5. IS Spatial Ability	13.45 (4.186)	.303 **	.155 *	.080	.285 **	-									
6. ID Spatial Ability	10.24 (3.771)	.181 *	−.008	.104	.121	.457 **	-								
7. ES Spatial Ability	12.42 (2.779)	.287 **	−.002	.112	.210 **	.518 **	.291 **	-							
8. ED Spatial Ability	16.83 (3.909)	.108	.081	.071	.117	.455 **	.354 **	.390 **	-						
9. Numerical Ability	14.68 (4.085)	.240 **	.087	.069	.213 **	.457 **	.302 **	.341 **	.366 **	-					
10. Arithmetical Ability	5.65 (2.343)	.242 **	.019	.117	.197 **	.445 **	.245 **	.334 **	.266 **	.824 **	-				
11. Geometrical Ability	7.39 (2.708)	.287 **	.117	.018	.239 **	.440 **	.301 **	.279 **	.334 **	.717 **	.643 **	-			
12. Total Mathematics Score	27.71 (8.309)	.279 **	.078	.071	.234 **	.494 **	.316 **	.353 **	.355 **	.953 **	.896 **	.855 **	-		
13. Verbal Intelligence Score	71.57 (15.162)	.046	−.119	.022	−.017	.359 **	.187 *	.308 **	.239 **	.565 **	.495 **	.454 **	.564 **	-	
14. Subjective PA	10.74 † (4.483)	.213 *	−.129	−.002	.029	.057	.071	−.023	.069	.030	.039	.073	.057	−.035	-
15. Objective MVPA	83.34 ‡ (40.300)	.122	−.043	.100	.073	.039	−.043	.024	−.012	.136	.139	.018	.108	−.067	.289 **

Note: subjective PA with all variables (*n* = 134) except objective MVPA (*n* = 102); objective MVPA with all variables except subjective PA (*n* = 137). † total number of hours spent undertaking physical activity during a typical week. ‡ average number of minutes spent undertaking MVPA per day. * *p* < .05, ** *p* < .01.

**Table 2:** Partial Spearman correlations between PA, FMS, spatial abilities, verbal intelligence, and mathematics in the whole sample (*n* = 182).

Variable	Mean (SD)	1	2	3	4	5	6	7	8	9	10	11	12	13
1. Total Locomotion	13.23 (1.753)	-												
2. Total Object Manipulation	10.10 (1.686)	.216 **	-											
3. Stability	2.58 (.880)	.260 **	.100	-										
4. Total FMS Score	25.91 (3.034)	.790 **	.676 **	.487 **	-									
5. IS Spatial Ability	13.45 (4.186)	.307 **	.213 **	.078	.312 **	-								
6. ID Spatial Ability	10.24 (3.771)	.176 *	.014	.102	.126	.425 **	-							
7. ES Spatial Ability	12.42 (2.779)	.287 **	.037	.111	.227 **	.459 **	.250 **	-						
8. ED Spatial Ability	16.83 (3.909)	.100	.113	.067	.125	.407 **	.325 **	.343 **	-					
9. Numerical Ability	14.68 (4.085)	.260 **	.188	.069	.269 **	.331 **	.242 **	.213 **	.288 **	-				
10. Arithmetical Ability	5.65 (2.343)	.253 **	.091	.122	.237 **	.330 **	.179 *	.220 **	.175 *	.759 **	-			
11. Geometrical Ability	7.39 (2.708)	.299 **	.193 **	.010	.278 **	.334 **	.248 **	.164 *	.261 **	.627 **	.540 **	-		
12. Total Mathematics Score	27.71 (8.309)	.308 **	.177 *	.072	.295 **	.379 **	.260 **	.229 **	.274 **	.931 **	.860 **	.815 **	-	
13. Subjective PA	10.74 † (4.483)	.215 *	−.134	−.001	.029	.075	.079	−.013	.080	.060	.065	.099	.094	-
14. Objective MVPA	83.34 ‡ (40.300)	.126	−.052	.102	.072	.067	−.031	.047	.005	.211 *	.199 *	.054	.178 *	.287 **

Note: subjective PA with all variables (*n* = 134) except objective MVPA (*n* = 102); objective MVPA with all variables except subjective PA (*n* = 137). † total number of hours spent undertaking physical activity during a typical week. ‡ average number of minutes spent undertaking MVPA per day. * *p* < .05, ** *p* < .01.

## Data Availability

The data presented in this study are openly available in the Loughborough University Repository, https://doi.org/10.17028/rd.lboro.24720804.
